# Pennsylvania Association of Dental Surgeons

**Published:** 1864-06

**Authors:** 


### PENNSYLVANIA ASSOCIATION OF DENTAL SURGEONS.

At a regular meeting of the Association, held on the evening of May 10th, 1864, the following named gentlemen were elected to serve as delegates to attend the American Dental Convention, to be held at Niagara Falls:

Dr. T. L. Buckingham, Philadelphia; Dr. E. Wildman, do.; Dr. J. H. Githens, do.; Dr. C. N. Peirce, do.; Dr. F. M. Dixon, do.; Dr. J. I. Griffith, do.; Dr. Geo. T. Barker, do.; Dr. R. A. Miller, Huntingdon, Pa.: Dr. Ely Parry,

Lock Haven, Pa.; Dr. Charles Moore, Pottstown; Dr. J. Hayhurst, ——; Dr. H. M. White, Philadelphia; Dr. James Truman, do.

After the transaction of the usual business, the subject for the evening's discussion, "Materials for Filling Teeth," was taken up, the opening remarks by

Dr. Peirce, who stated the principal objects in filling teeth were to prevent further disintegration, and supply the parts destroyed, thus retaining their efficiency in articulation, mastication, &c.

The materials used, in order to accomplish these results, must possess the following properties, viz: malleability, adaptability, indestructibility, adhesiveness and density. The necessity of these qualities must be patent to all, when they remember that the fluids of the mouth, when in a vitiated condition, are the destructive agents, or exciting cause of decay, and that the dentine, or tooth-bone, can only be preserved by a thorough and complete adaptation of the filling to the walls of the cavity filled. The material in which these properties are most nearly blended is gold foil, which meets with universal approbation in a vast majority of cases. Where economy is an object to the patient, tin should be substituted, as it approximates nearer those invaluable properties found in gold, than any other substance. The fluids of the mouth have no effect upon it. Its density being less than that of gold, renders it *less efficient* in cavities upon *surfaces*, where it is exposed to attrition in mastication. But in frail teeth, approximal cavities, and teeth affected by white decay, when its color is not objectionable, its preserving qualities are equal, if not superior, to those of gold. Compared with amalgams and silver—were it practicable to use the latter—it is far more efficient.

A substance much used and highly lauded by empirics, is os-artificial, which has no merit for a permanent filling, and should never be used for a temporary one, where the tooth retains its vitality. Its escharotic effect, as well as its ready dissolution, renders it not only inefficient but, in some cases, absolutely injurious.

The doctor had treated two teeth with Wood's metal, as an experiment, several months since, and upon a recent examination, one retained its original appearance, while the other was covered with small pits, which he attributed to imperfect manipulation.

Dr. Buckingham said that all admitted that gold was the best material for filling teeth, and next to tin foil, which he thought was not used as much as it should be. Many teeth that are now filled with very large gold plugs, he thought, could be better filled with tin. The density of gold being greater than tin rendered it more difficult to pack in inconvenient situations where the cavity was large. Much time and hard work was consumed in filling such teeth. A tooth of this class might occasionally be filled, to show that gold could be used as well in these as in smaller cavities. The class of teeth he thought best adapted for the use of tin were the molars that have been diseased for a long time. A good healthy tooth, with the nerve unexposed, or where it has been exposed in excavating, should always be filled with gold. He could refer to a number of cases of diseased teeth, that had been under treatment for a long time, until they were supposed to be in a healthy condition, then filled with good solid gold fillings, but a short time elapsed before it was found necessary to extract them. Such teeth might be filled with tin with less labor, and much less expense to the patient, and last as long as they would filled with gold. A tin filling will preserve a tooth as well as a gold one, and in some cases, he thought, better. There seemed to be an affinity between the tin and the tooth that protected each other. Very imperfect tin fillings will sometimes preserve teeth, where, if gold had been used, they would have been lost in a short time. This he had particularly noticed in children's teeth.

Amalgam he used only in extreme cases. He had seen teeth filled with it and preserved a number of years, but as a general rule they soon begin to break away around the plug. There appears to be a chemical action between the material and the tooth, by which the latter is decomposed and the plug oxidised. He had formerly used amalgam to a greater extent than he now does. It takes several years to tell the merits of a material for filling teeth. Cases frequently occurred where the teeth were so frail that the choice was between filling with some plastic material or extraction. In such cases, when the molars and bicuspids were the teeth to be treated, he thought the use of amalgam perfectly justifiable. He preferred it to osteo-plastic, which he never used, except in cases of sensitive dentine, and then only allowed it to remain a few days to destroy the sensibility. Wood's fusible metal he had not tried, and therefore could not say anything in regard to it.

Dr. Dixon said, that though physically unable to speak, his great interest in the subject brought him to give his opinion.

In his judgment, the only material that could universally be relied on for filling teeth was gold foil. All others had some objections, and some were wholly unfit for the purpose. He was satisfied many operators were not aware of the extent to which the use of gold foil could be carried in teeth that had been doomed as unfit for any thing but the plastic materials. He was decidedly opposed to experimenting on teeth to the extent many were in the habit of doing. Patients expect, and should receive from our hands our best efforts. If we make an indiscriminate use of the plastic material in filling these teeth, we not only do them a great wrong, but bring lasting discredit upon the profession.

In his practice he had confined himself almost exclusively to gold foil. His experience in the use of oxy-chloride had been very limited; but that, in connection with an extended observation of the work of other operators, had convinced him that it was inadmissible as an agent for filling teeth, and he was satisfied great injury was being inflicted by its use. In regard to tin foil he desired to give a clear and decided opinion to its value as a material for filling teeth. The greatest objection to it, he thought, was found in its soft character, unfitting it for fillings upon the masticating surface; but in all others, it answered fully his expectations in preserving the teeth. Where expense was a consideration with the patient, it was of great value.

Dr. Barker thought he understood Dr. Dixon's meaning in opposing experimenting. He did not think he wished to be understood as objecting to a proper effort to test different substances brought to the notice of the profession, but the indiscriminate use of articles that have not been thoroughly tested by careful observation. With this view of the case, he could accord with him.

He had used the oxy-chloride to some extent, but had found that, with all the care he was capable of bestowing, it would dissolve. In some mouths, this process was carried on with greater rapidity than in others. He had used a preparation of gutta percha dissolved in chloroform, to keep the filling dry, covering it carefully, and allowing it to remain as long as deemed necessary.

The time had not been sufficient since the introduction of Wood's metal to speak positively in regard to it; but so far as he had used it, it had resulted very satisfactorily. One filling that he recently examined, after six months trial, still retained all its original lustre and general healthy appearance.

Dr. Wildman had made an experiment to test the relative fusibility of Wood's metal marked No. 1, and a piece compounded from Lipowit's analysis (Bi. 15, Pb. 8, Sn. 4, Cd. 3), of one of Wood's compounds, the melting of which is given to be 140° Fa. The two metals were placed in water on a wire gauze diaphragm, the bulb of a thermometer was also in contact with the diaphragm and near the metals. The heat was gradually raised and the effect noted. Wood's No. 1 became pasty at a 165° Fa., and remained in that condition up to 176° Fa., at which temperature it became fluid and ran through the diaphragm. The compound made according to Lipowit's analysis became pasty at 163° and was fluid and ran through at 165.° This compound was slightly acted upon by pure sulphuric acid, energetically by nitric, leaving a dark stain, and feebly by hydro-chloric acid, *leaving a bright surface.*

The advantage that Wood's No. 1 possesses is, that it remains plastic through a greater range of temperature, thereby possessing a property rendering it better adapted for filling cavities solid, by being less liable, from a slight change of temperature, *to creep* from the walls of the cavity. He had never used any of the fusible metals for filling teeth. He had not any experience in the use of os-artificial, and had no faith in it from its first introduction; and from what he had seen of it, his first impressions had been fully confirmed.

Dr. Fouche said great discrimination was needed in selecting materials for filling teeth. He regretted that so large an amount of manual labor was required to condense gold foil in cavities of the teeth, which labor, when continued for eight or nine hours a day for successive days, months and years, is attended with great physical exhaustion to the operator. Although gold was his first choice of the metals for filling teeth, nevertheless, there was no salvation for a tooth in the mere fact of its having gold in it. That must depend upon the manner in which the gold is worked within the cavity, and how nearly it is made to represent the tooth both in density and form. The most commendable feature of gold is its entire freedom from the decomposing effects of the oral secretions. On the other hand, the many excessively large cavities, after the extirpation of the nerve—the pale and delicate condition of devitalized teeth—the difficulty of access to many cavities, rendering it difficult to make a perfectly solid filling—the physical and pecuniary considerations affecting the patients, are sufficient reasons why gold foil should not be used in all cases, and why at times we should discriminate in favor of tin foil, amalgam, oxy-chloride, Hill's Stopping, &c., &c.

When cost of material was of importance to the patient, he would use tin foil, but only in the back teeth. For large molar cavities, and when it is desirable to build up the form of the tooth with all its cusps, he would use Townsend's Amalgam.

In filling devitalized, discolored and frail front teeth, he prefers the oxy-chloride of zinc, as it imparts a more natural hue to the tooth. By excluding the moisture for the space of an hour, which he accomplishes by covering the filling with wax, it becomes very hard, and the patient is better satisfied with its appearance, though it may be found necessary to renew it every two years. For the reasons given he could not agree with the wholesale denunciations of the plastic materials.

The subject was, on motion, continued for discussion at the next meeting of the Association. Adjourned.

